# Protocol to maintain single functional mouse hematopoietic stem cells *in vitro* without cell division

**DOI:** 10.1016/j.xpro.2021.100927

**Published:** 2021-11-01

**Authors:** Miriam Belmonte, David G. Kent

**Affiliations:** 1 Wellcome MRC Cambridge Stem Cell Institute, University of Cambridge, Hills Road, Cambridge CB2 0XY, UK; 2 Department of Haematology, University of Cambridge, Cambridge CB2 0XY, UK; 3 York Biomedical Research Institute, Department of Biology, University of York, York YO10 5DD, UK

**Keywords:** Cell culture, Single Cell, Flow Cytometry/Mass Cytometry, Immunology, Microscopy, Stem Cells

## Abstract

This protocol details the isolation and *in vitro* maintenance of single hematopoietic stem cells (HSCs) in the absence of the bone marrow niche. The HSCs do not divide over 7 days and fully retain their long-term functional capacity in transplantation assays. Following hibernation culture, HSCs can be used to study quiescence exit and can be genetically manipulated as single cells prior to division.

For complete details on the use and execution of this protocol, please refer to Oedekoven et al. (2021).

## Highlights

• *In vitro* maintenance of single HSCs in the absence of a hematopoietic niche
• *In vitro* system to study quiescence exit in single HSCs
• Ability to manipulate HSCs via gene modification at the single-cell level

## Graphical abstract

**Figure:**
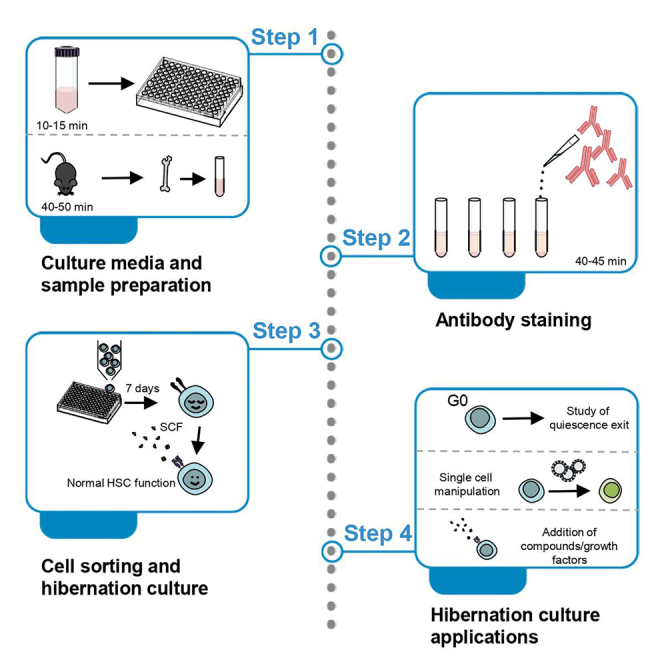

## Before you begin

Hematopoietic stem cells are thought to reside at the apex of the hematopoietic hierarchy and possess the unique capacity to both self-renew (perpetuating themselves as an undifferentiated population) and differentiate (generating the correct numbers and types of mature cell progeny to perform the necessary functions of the blood system). The development of clonal approaches to purify blood stem and progenitor cell populations has enabled significant breakthroughs in the field including the formal documentation of HSC functional heterogeneity and the development of a wide array of molecular tools to study HSCs at the single cell level. This has also led to a range of studies aimed at mimicking the HSC environment *in vitro* in order to develop protocols that would successfully expand hematopoietic populations in a dish.

In Oedekoven et al., we described a powerful culture system which maintains mouse and human HSCs as single cells, while preserving their functional and molecular properties, allowing a 7-day culture window to study HSCs in a distinct physiological context (Oedekoven et al., 2021). These “hibernation cultures” keep HSCs from dividing by removal of key mitogens SCF and TPO from standard conditions. Optimization and utilization of such *in vitro* cultures would have a range of applications for manipulating HSCs at the single-cell level in both experimental and clinical research.

The protocol below describes the specific steps for using serum-supplemented medium. However, we have also validated the hibernation protocol in the serum-free culture media published in Wilkinson et al. (2019) as reported in Oedekoven et al. (2021).

### Prepare washing buffer (PBS 2%FCS)

**Timing: 10 min**

1. Mix all reagents listed below:

**Table:** 

Reagent	Final concentration	Amount
PBS	-	490mL
Fetal Calf Serum	2%	10mL
**Total**	**N/A**	**500mL**

2. Filter using a 0.2μm filter and store at 4°C for up to 1 month

### Prepare culture medium

**Timing: 10 min**

3. To prepare 10 mL of medium (amount for loading 100μL/well of a 96-well plate), mix all the reagents listed in the table below except for IL-11 (which will be added to the plate after sorting the cells)

**Table:** 

Reagent	Stock concentration	Final concentration	Amount (mL)
StemSpan SFEM	-	-	8.78
Fetal Calf Serum	-	10%	1
Penicillin / Streptomycin	100 units/mL	1%	0.1
L-Glutamine	200mM	1%	0.1
10^−4^M 2-Mercaptoethanol	-	0.2%	0.02
			
Interleukin 11 (IL-11)	10ng/mL	20 ng/mL	0.02

4. Filter the medium using a 0.2μm filter before use
  a. Store the medium at 4°C for up to 1 week, if needed

### Pre-load medium a U-bottom 96-well plate

5. Load 50μL of the culture media (described above) per well into U-bottom 96-well plate(s).

**CRITICAL:** Keep the rest of the medium at 4°C as this will be added after the cell sort using a 2X concentration of IL-11. This is to avoid the wastage of recombinant cytokines in the event that there is a technical failure at the cell sorter and to synchronize the stimulation time of the cytokine).

## Key resources table

**Table:** 

REAGENT or RESOURCE	SOURCE	IDENTIFIER
**Antibodies**		

Anti-mouse CD45 FITC (dilution 1:100)	BD Biosciences	clone 30-F11
Anti-mouse EPCR PE (dilution 1:100)	STEMCELL	clone RMEPCR1560
Anti-mouse CD150 PE-Cy7 (dilution 1:100)	Biolegend	clone TC15-12F12.2
Anti-mouse CD48 APC (dilution 1:100)	Biolegend	clone HM48-1
Anti-mouse Sca-1 BV421 (dilution 1:200)	Biolegend	clone D7

**Chemicals, peptides, and recombinant proteins**		

PBS	Sigma	Catalog #D8537
Fetal Calf Serum (FCS) *FCS should be screened for high efficiency haematopoietic colony growth*	Sigma	Catalog #F7524
StemSpan	STEMCELL Technologies	Catalog #09650
Ammonium Chloride (NH_4_Cl)	STEMCELL Technologies	Catalog #07850
2mM L-Glutamine	Life Technologies	Catalog #25030081
2-Mercaptoethanol	Fisher Scientific	Catalog #11528926
Penicillin and Streptomycin (Pen/Strep)	Sigma	Catalog #P4333
7-Aminoactinomycin D (1mg/mL solution in DMSO)	Life Technologies	Catalog #A1310
Recombinant mouse IL-11	Bio-Techne	Catalog #418-ML

**Critical commercial assays**		

EasySep Mouse Hematopoietic Progenitor Enrichment kit	STEMCELL Technologies	Catalog # 19856
EasySep Magnet	STEMCELL Technologies	Catalog # 18000

**Experimental models: Organisms/strains**		

C57BL/6 mice, 8–12 weeks old	Charles River Laboratories	N/A

**Other**		

Dissecting instruments (scissors and forceps)	N/A	N/A
Mortar and pestle	N/A	N/A
50μm filters	Wolf Laboratories	Catalog #04-0042-2317
5 mL polypropylene tubes	VWR International	Catalog #352063
5 mL polystyrene tubes	Scientific Laboratory Supplies	Catalog #352054
50 mL conical tubes	VWR International	Catalog #525-0402
U-bottom 96-well plates	Scientific Laboratory Supplies	Catalog #353077

## Step-by-step method details

### Mouse bone marrow cell preparation

**Timing: approximately 2–3 h, depending on the number of animals/samples**

This section describes the preparation of murine bone marrow cell suspension and magnetic bead depletion of progenitor cells (optional) by negative selection using the EasySep™ Mouse Hematopoietic Progenitor Cell Enrichment Kit (STEMCELL).

1. Harvest the bone marrow cells from femora, tibiae and pelvic bones removed from the mice by crushing the bones in 10mL PBS 2%FCS using a mortar and pestle. For specific animals where maximal cells are required, BM can also be obtained from the spine and forearms
2. Transfer the cell suspension into a 50 mL conical polypropylene centrifuge tube
3. Centrifuge the cell suspension for 5 min at 300 x g at room temperature
4. Remove the supernatant and resuspend the cells in 3mL PBS/FCS
5. Lyse the red blood cells by adding 5 mL Ammonium Chloride (NH_4_Cl, STEMCELL) incubating the cells on ice for 10 min, vortexing after the first 5 min
6. Fill the tube up to 50mL with PBS/FCS and centrifuge for 5 min at 300 x g at room temperature
7. Resuspend the cells in 500μL/mouse PBS/FCS and transfer into a polystyrene round-bottom tube
  a. Add 1mL of PBS/FCS to the empty 50mL conical polypropylene centrifuge tube from step 6 and use these leftover cells for preparing control samples of non-depleted cells. This is important to assist the user in setting gates for sorting as well as for determining the absolute frequencies of subpopulations of interest, since these cannot be measured from the enriched sample. Filter the suspension using a 50-μm filter. Use 100μL of this suspension into each of the FACS control tubes (unstained, 7AAD only, FITC only, PE only, APC only, PE-Cy7 only, BV421 only)
    ***Optional:*** Perform lineage depletion by using EasySep mouse hematopoietic progenitor cell enrichment kit (STEMCELL). The lineage depletion step will reduce sorting time and reagent costs, especially when isolating HSCs from multiple mice. We typically use the Hematopoietic Enrichment Cocktail and EasySep technology from STEMCELL Technologies, however, other commercial suppliers of lineage depletion kits exist. Alternatively, the lineage depletion step could by replaced using directly conjugated antibodies which allow the exclusion of the differentiated population during the cell sorting (CD5, CD11b, CD19, CD45R/B220, Ly6G/C(Gr-1), TER119). This, however, will increase sorting time and reagent costs, compared to performing the lineage-depletion step prior to cell sorting.
    Manufacturer protocol was followed with minor modifications to prioritize yield over purity in this pre-sort “debulking” step:
    i. Add 10μL/mouse EasySep Mouse Hematopoietic Progenitor Cell (HSPC) Isolation cocktail and incubate for 15 min, on ice.
    ii. Add 15μL/mouse EasySep Streptavidin RapidSpheres and incubate for 15 min on ice
    iii. Add PBS/FCS to a total of 2.5mL
    iv. Insert the tube into the EasySep Magnet and incubate for 3 min at room temperature
    v. Transfer the supernatant into a new tube and repeat the magnet incubation step one additional time
    vi. Centrifuge the cell suspension for 5 min at 300 x g at room temperature
    vii. Resuspend the cells in 100μL/mouse PBS/FCS and transfer into a polypropylene tube to perform antibody staining

### Antibody staining

**Timing: 40–50 min**

This section describes the incubation of bone marrow samples with fluorescence-tagged antibodies and the preparation of compensation controls.

8. Add the following antibodies to the FACS control tubes:

**Table:** 

Reagent	Label	Dilution
CD45	FITC	1:100
CD48	APC	1:100
CD150	PE-Cy7	1:100
EPCR	PE	1:100
Sca-1	BV421	1:200

9. Add all antibodies at the concentration specified above to the sample tubes (making an antibody master mix is recommended if more than one sample)
10. Vortex the cells in each tube and incubate on ice in the dark for 30 min
11. Wash the cells by adding 1mL of PBS/FCS to each tube and centrifuging for 5 min at 300 x g at room temperature
12. Resuspend the control tubes in 300μL PBS/FCS and add 1:1000 7AAD to the 7AAD-only control tube
13. Resuspend the sample tube in 500μL/mouse 1:1000 7AAD in PBS/FCS and filter the suspension using a 50-μm filter into a new polypropylene tube. Add 500μL 1:1000 7AAD in PBS/FCS to the original tube to make sure to rinse out the remaining cells and transfer them to the new tube through the 50-μm filter

### Sorting of ESLAM Sca-1^high^ LT-HSCs

This section describes the workflow to isolate single HSCs using fluorescent-activated cell sorting (FACS).

14. Use the unstained control to set voltages of each channel on the flow sorter and use 7AAD-only control to set the viability gates. Use the single stain controls to set up the compensation for each detection channel

***Optional:*** Compensation beads can substitute for single stain controls, but it is important to remember that they will not account for background staining or autofluorescence and it is advisable to check the full panel in an initial setup phase.

15. Apply the compensation settings to the sample tubes
16. Load the sample tube on the flow sorter and set gates as shown in Figure 1

**Figure 1:**
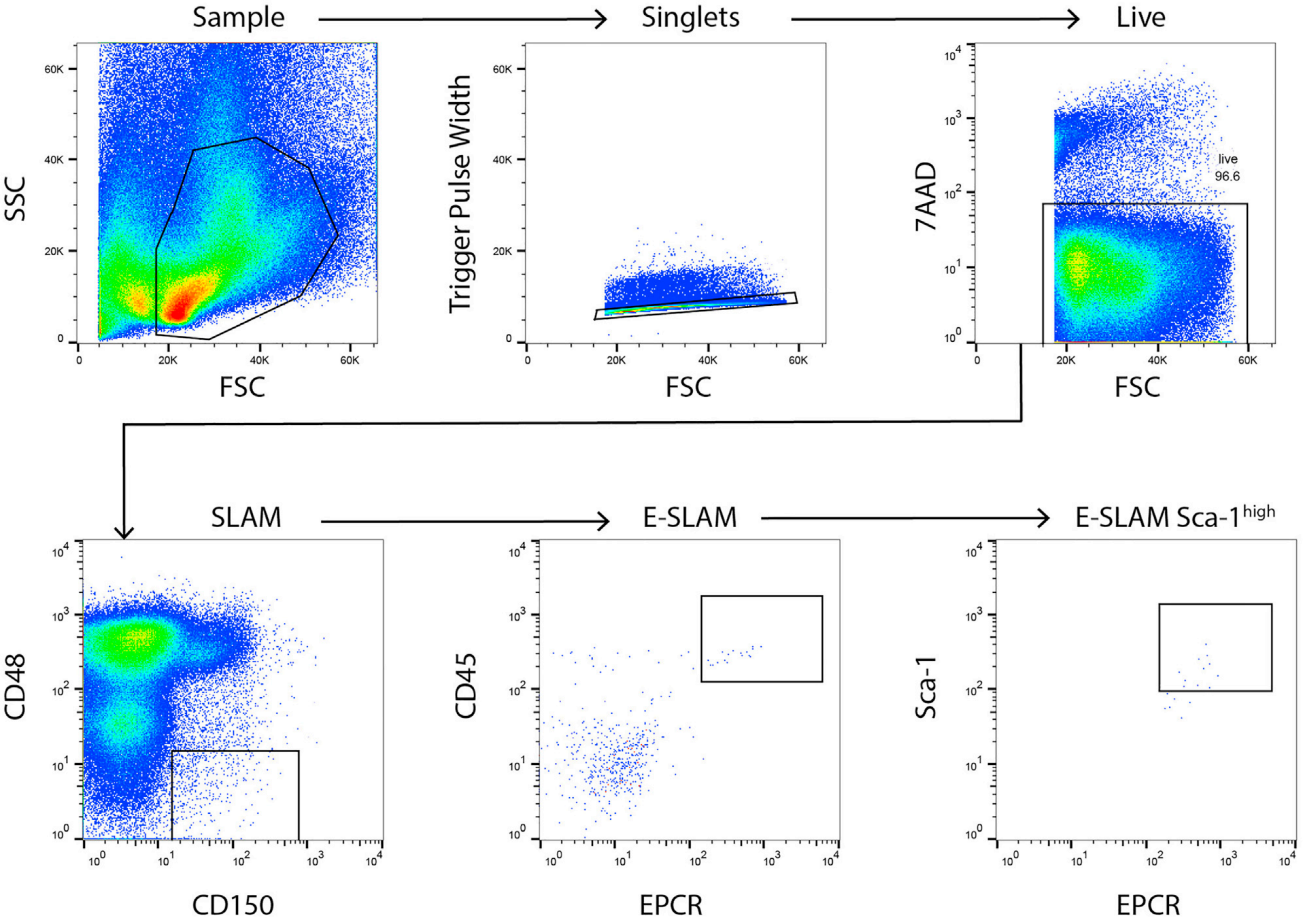
Representative gating strategy to purify ESLAM Sca-1^high^ LT-HSCs On a SSC-A vs FSC-A plot, gate a region which excludes most of the dead cells and debris and very large cells. Gate on single cells using pulse width and FSC-A as shown. Exclude dead cells by gating 7AAD-negative cells. To set the SLAM population, gate CD48^-^ CD150^+^ cells, being careful not to go too high into the CD48^dim^ population. From the SLAM population, gate CD45^+^EPCR^+^cells with a relatively strict EPCR^+^ gate (initially described as EPCR^++^)(Kent et al., 2009). To set the ESLAM Sca-1^high^ population which has an increased proportion of LT-HSCs but reduced overall yield, gate Sca-1^high^ cells (described in Wilson et al., 2015).
17. Use the single-cell deposition unit of the sorter to place 1 cell into each of the wells of U-bottom 96-well plates, each well having been preloaded with 50μL media (described above in “Before you begin” section)
18. Load 50μL media (described above) supplemented with a 2X cytokine concentration to bring the final medium to 1X cytokines

**Pause point:** The cell suspension can be stained for longer than 30 minutes on ice, but it is recommended not to exceed ∼1hour in order to avoid overstaining the samples and increasing background signal. For a quicker preparation of single stain controls, tubes can be incubated for a shorter time (∼15 minutes) at room temperature, but this is not advisable for primary samples where room temperature incubation may alter the function of cells.

***Optional:*** Given the long preparation time required to obtain cell suspension pre-sorting, bone marrow harvest can be performed the day before the sort and the cell suspension can be stored in 10%FCS/PBS at 4°C overnight. If considering this option, consider storing the sample in a larger liquid volume (∼5mL/mouse) and then spinning at 300 x g for 7 minutes followed by addition of crude DNase I to the pellet in the morning before undertaking lineage depletion and/or antibody staining described above.

## Expected outcomes

### Mouse bone marrow cell preparation

Cell suspension obtained from normal mice should look pinkish and be a bit cloudy. After the first centrifugation step the cell pellet will look reddish, but following the RBC lysis step and centrifugation, the supernatant should look clear and the pellet will be whitish, with red edges.

### Preparation of cells for cell sorting

The final cell suspension should be contained in a volume of ∼1mL/mouse processed and filtered before sorting, as described above, to remove any clumps of cells which might clog the flow instrument. When resuspended in 2%FCS/PBS, the cell suspension should look translucent.

### Sorting of ESLAM Sca-1^high^ LT-HSCs

Representative flow cytometric plots for a typical ESLAM Sca-1^high^ cell sorting procedure are shown in Figure 1. Viable cells are gated based on their FSC and SSC properties and their exclusion of 7AAD. CD48^+^ cells are excluded by gating the bottom of the three major populations. These cells are then visualized in a CD45/EPCR plot and the double positive cells are selected. CD150^+^CD48^-^EPCR^+^Sca-1^high^ cells are then selected, which represents ∼0.003% of the whole bone marrow cells in an adult C57BL/6 mouse.

### Single-cell culture of HSC

**Figure 2:**
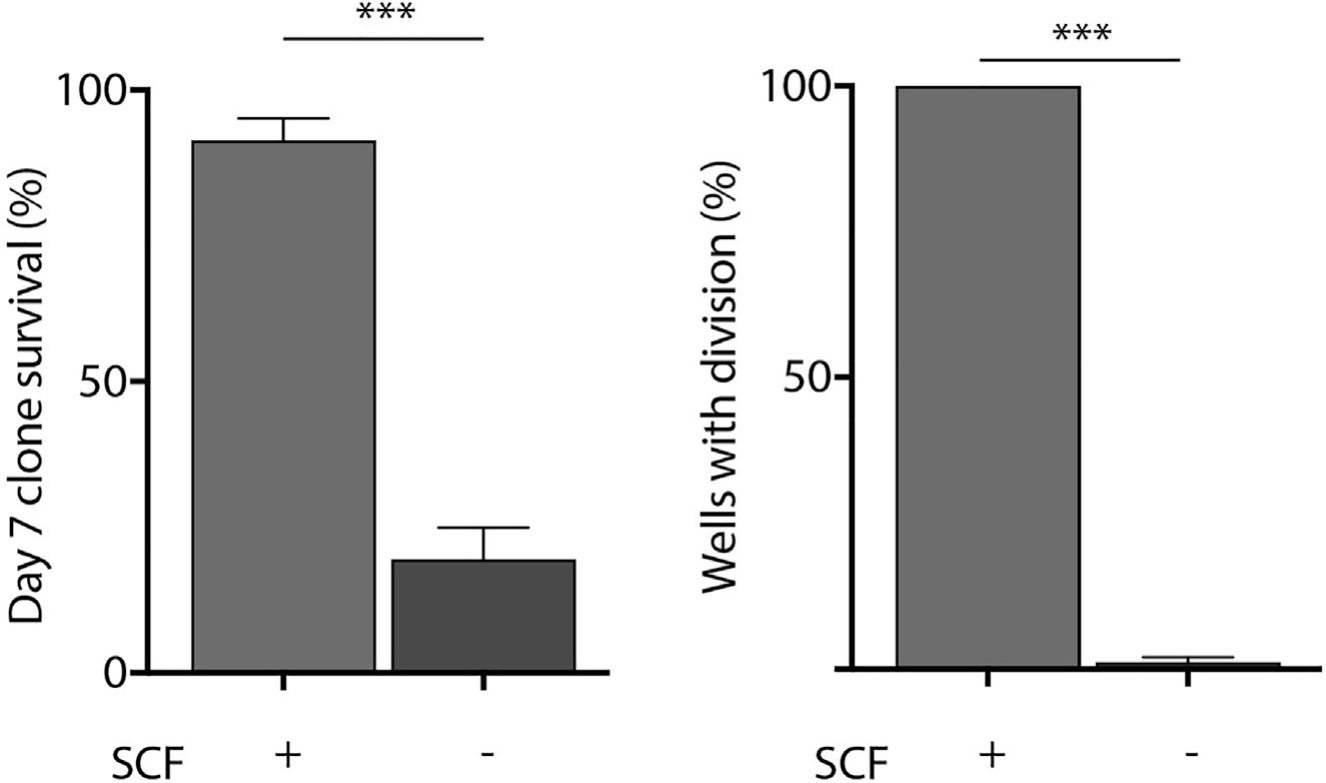
Cells in hibernation conditions have reduced survival and do not divide Adapted from Oedekoven et al. (Oedekoven et al., 2021). Left panel: HSC survival in hibernation conditions is decreased compared to SCF-supplemented medium (+SCF n=355, 5 biological replicates; -SCF n=1722, 7 biological replicates). Right panel: number of clones that had divided (numbers of wells with >2 cells were scored to determine) at day 7 post-isolation. Only culture conditions without SCF retained HSCs as single cells. Bars show mean with SEM. Unpaired t-test: ∗p<0.05, ∗∗p<0.01, ∗∗∗p<0.001.

**Figure 3:**
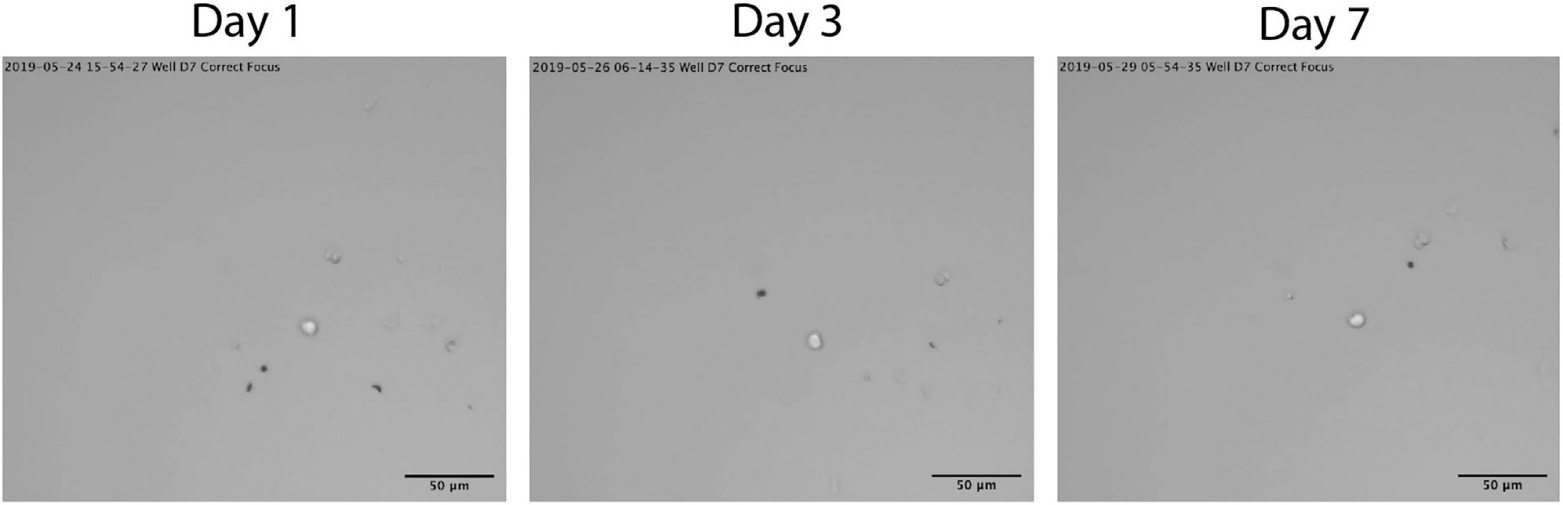
Time-lapse imagine of single HSCs in hibernation cultures The three pictures show an individual well imaged on day 1, 3 and 7 of culture in the hibernation condition. Single cells do not divide over the course of the 7-day hibernation culture. Scale bar represents 50um. See also the supplemental video in Oedekoven et al. (Oedekoven et al., 2021)

In order to visualize the cells under the microscope, adjust the field of view to look at the entire well, then focus on the center of the well by using the coarse adjustment. In 96 well U-bottom plates, this is where the cell typically settles a few hours after sort. To facilitate the visualization of the single cells under the microscope, the plate can be spun in a centrifuge for 5 min at 240 x g to allow the cells to be collected at the center of the well (cells may not appear as spherical immediately after spinning). Alternatively, to facilitate visual identification, the first well can be used to sort 100 viable cells to allow establishment of the field of view for subsequent wells. Between 20 and 40% of single cells will survive 7 days of culture in the hibernation condition, compared to ∼98% survival in 300ng/mL SCF-supplemented media. ∼99% of the input HSCs cultured in the hibernation condition will not divide during the 7-day culture period and will be maintained as single cells (Figures 2 and 3).

### Single cells for future manipulation

In Oedekoven et al., 2021, we showed that single HSCs could have virus delivered to them without undergoing division (Oedekoven et al., 2021). However, there are a wide range of other studies that could be imagined, including monitoring symmetric versus asymmetric partitioning of cellular components using high resolution imaging (Filippi, 2021; Wang et al., 2021), single cell immunostaining (Ema et al., 2007), or screening of compounds for improved survival or induction of cell division.

**Figure 4:**
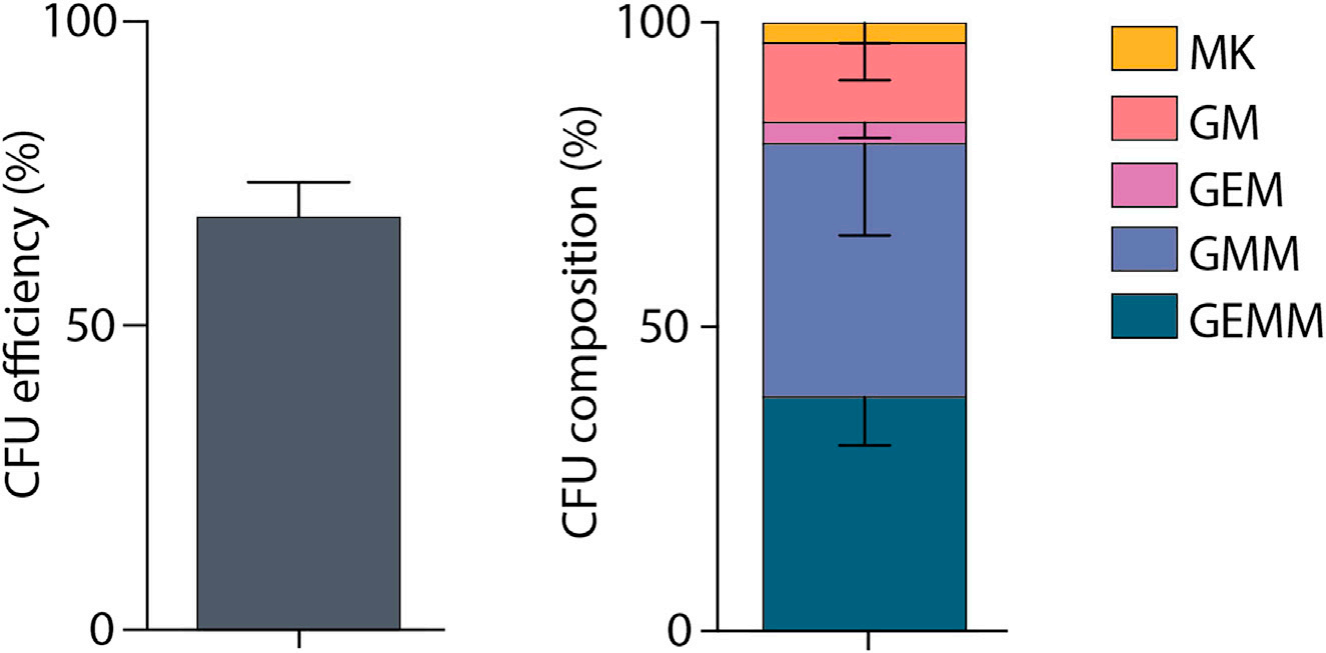
Single HSCs in hibernation culture retain multi-potency Adapted from Oedekoven et al. (Oedekoven et al., 2021). Left panel: Colony forming cell assay efficiency for single HSCs in hibernating culture (n=230, 6 biological replicates). Right panel: ∼80% of single cells generate colonies of at least three lineages in colony forming cell assays (n=166, 3 biological replicates). Colonies are defined as MK (containing cells positive for CD41, a megakaryocyte marker), GM (containing cells positive for granulocyte/monocyte markers Gr-1 and CD11b), GEM (containing cells positive for granulocyte/monocyte markers Gr-1 and CD11b, GMM (positive for GM and MK markers), and GEMM (positive for GM, MK and erythrocyte markers). Bars show mean with SEM.

60–70% of single HSCs cultured in the hibernation condition for 7 days are able to generate clones in colony forming cell assays at day 14, with the majority generating at least three different mature cell types (as determined by flow cytometry for cell surface markers Ly6g (Gr-1), CD11b (Mac-1), Ter-119 and CD41) (Figure 4).

## Limitations

### Low survival in hibernation culture

HSC survival in hibernation culture is decreased in the absence of SCF compared to SCF-supplemented medium, as shown in Figure 2. Further optimization of the hibernation culture and/or the addition of additional supplement (e.g., Rho kinase inhibitors (Claassen et al., 2009), fibronectin, PVA (Wilkinson et al., 2019)) could improve the survival *in vitro* and allow larger-scale studies, without requiring the use of larger number of animals to obtain higher cell output.

### Potential selection of a specific HSC phenotype

In Oedekoven et al. (Oedekoven et al., 2021), we investigated whether differences in HSC subtypes (in accordance with Dykstra et al., 2007) were observed between hibernating and freshly isolated HSCs in single cell transplantation experiments. Although it would need to be confirmed in a larger number of recipients, the current data suggest that hibernation conditions might preferentially retain α-HSC (Oedekoven et al., 2021) which is also consistent with the higher levels of CD150 expression previously reported (Beerman et al., 2010; Morita et al., 2010). This could indicate that the α-HSC subtype is more resilient in the hibernation culture and that the cytokine deprivation might be selecting for the more myeloid-biased HSCs and this property may also be related to the delayed engraftment that is observed in α-HSCs.

### Long-term survival of HSCs in hibernation culture

**Figure 5:**
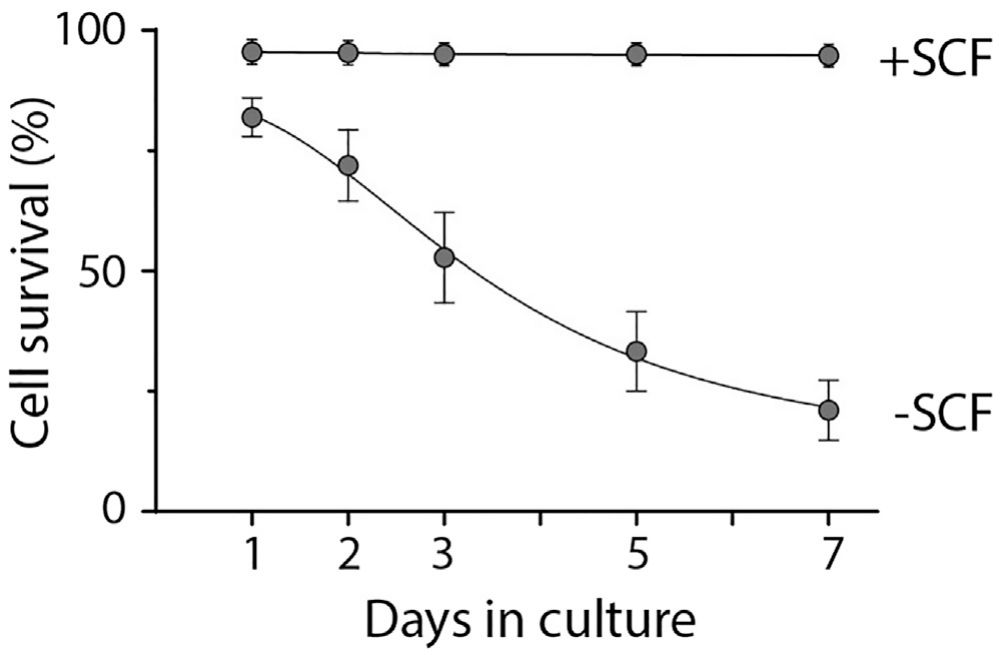
Time course of survival over 7 days of SCF deprivation SCF-deprivation decreased HSC survival in culture compared to SCF-supplemented conditions, and only ∼25–35% remain viable single cells up to 7 days in culture (+SCF, 5 biological replicates; -SCF, 5 biological replicates). Bars show mean with SEM.

Cell survival decreases overtime, and reduces after 7 days of culture, making longer term cultures in this current condition challenging (Figure 5). We have tested up to 10 days in some experiments without observing an additional decrease in cell survival, however, we are unable to say whether extended culture beyond 10 days would also permit the same subset of HSCs to survive.

### Proliferation of HSCs in hibernation culture

Our data show that over 99% of HSCs cultured in hibernation condition remain as single cells over the 7-day hibernation culture. However, a small fraction of single cells (∼0.8%) do undergo division. In order to confirm that none of the cells which survived on day 7 have undergone division followed by death of one daughter cells, we recommend performing daily counts or time-lapse imaging to monitor potential cell division/cell death.

### Gene expression changes

Gene expression profiling of single LT-HSCs in 7-day hibernation conditions revealed a high retention of G0/G1 cell cycle genes, as well as self-renewal regulators of the HSC-related MolO signature (Wilson et al., 2015), suggesting that such genes are indeed likely to be essential for HSC function and confirming the functional HSC identity of hibernating HSCs at the transcriptional level. However, some molecular differences between hibernating and freshly isolated HSCs were observed, and a number of regulators were not expressed in hibernating HSCs (e.g., members of the AP1 complex). These observations suggest that the hibernation cultures might drive the reduced expression of these factors. That said, it is important to note that SCF-stimulation of the hibernating HSCs did not re-initiate their expression, potentially disfavoring this hypothesis (Oedekoven et al., 2021).

## Troubleshooting

### Problem 1

Insufficient time on the sorter to obtain the required cell number and/or insufficient number of cells obtained after sorting.

### Potential solution

Insufficient time/cells might be caused by insufficient number of input cells. Consider harvesting more bone marrow (more mice) and performing the optional pre/sort lineage depletion (e.g., using EasySep Lineage Depletion cocktail from STEMCELL Technologies). This step will help decreasing the number of contaminating cells and will increase the absolute yields. If performing single cell sorting, it is advisable not to increase the speed of cell sort beyond ∼2000 cells/s in order to avoid too many aborted decisions in the single cell/droplet setting on the flow sorter.

### Problem 2

High number of 7AAD-positive cells at the sorter.

### Potential solution

Depending on the final panel of antibodies/fluorochromes used, there may be some compensation issue causing cells to appear 7AAD positive. This is most likely to be the case if these cells are directly above the viable cell fraction in FSC (as events lower in FSC are likely to be dead). Check with a flow operator and adjust the flow cytometer’s settings and compensation where necessary. If the cells were indeed dead, some possible approaches can be taken including keeping the cells and plates on ice and not leaving cells overnight. It would also be worth checking reagents to ensure that they are at the correct concentrations and not inadvertently driving cell lysis (e.g., 10× PBS versus 1× PBS).

### Problem 3

Difficulty finding the single cells in individual wells under the microscope.

### Potential solution

There are two possible issues: 1) the cells were not sorted properly or 2) the cells are present, but you cannot find them. If the cells were not sorted correctly in the wells, check with the flow operator the flow cytometer’s settings and calibration used (including sort reports) and consider performing a set of optimization experiments with cells (not beads) to ensure that the single cell deposition unit is functioning properly. If the cells are there, but you cannot find them, another possible explanation is that the medium is full of debris which make hard to distinguish the cells. We recommend always filtering the media before sorting into it, by using a 0.22μm filter and to sort ∼100 viable cells into well A1 to allow efficient finding of the focal plane for visualizing cells. We also suggest to always prepare a plate loaded with media (as above) with 300ng/mL Stem Cell Factor (SCF), as good control for the sorter/sorting. In this latter scenario, we would expect to obtain over 90% survival and cell division to occur following 24–48 h (Kent et al., 2013).

## Resource availability

### Lead contact

Further information and requests for resources and reagents should be directed to and will be fulfilled by the lead contact, David G. Kent david.kent@york.ac.uk.

### Materials availability

This study did not generate new unique reagents.

## Data Availability

The data sets supporting this protocol, and used in Figures, have not been deposited in a public repository but are available from the corresponding author upon request.

## References

[bib1] Beerman I. (2010). Functionally distinct hematopoietic stem cells modulate hematopoietic lineage potential during aging by a mechanism of clonal expansion. Proc. Natl. Acad. Sci. U S A.

[bib2] Claassen D.A., Desler M.M., Rizzino A. (2009). ROCK inhibition enhances the recovery and growth of cryopreserved human embryonic stem cells and human induced pluripotent stem cells. Mol. Reprod. Dev..

[bib3] Dykstra B. (2007). Long-term propagation of distinct hematopoietic differentiation programs in vivo. Cell Stem Cell.

[bib4] Ema H. (2007). Adult mouse hematopoietic stem cells: purification and single-cell assays. Nat. Protoc..

[bib5] Filippi M.D. (2021). Hematopoietic stem cell (HSC) divisional memory: the journey of mitochondrial metabolism through HSC division. Exp. Hematol..

[bib6] Kent D.G. (2009). Prospective isolation and molecular characterization of hematopoietic stem cells with durable self-renewal potential. Blood.

[bib7] Kent D.G. (2013). Self-renewal of single mouse hematopoietic stem cells is reduced by JAK2V617F without compromising progenitor cell expansion. PLoS Biol..

[bib8] Morita Y., Ema H., Nakauchi H. (2010). Heterogeneity and hierarchy within the most primitive hematopoietic stem cell compartment. J. Exp. Med..

[bib9] Oedekoven C.A. (2021). Hematopoietic stem cells retain functional potential and molecular identity in hibernation cultures. Stem Cell Reports.

[bib10] Wang W. (2021). Cytokine combinations for human blood stem cell expansion induce cell type- and cytokine-specific signaling dynamics. Blood.

[bib11] Wilkinson A.C. (2019). Long-term ex vivo haematopoietic-stem-cell expansion allows nonconditioned transplantation. Nature.

[bib12] Wilson N.K. (2015). Combined single-cell functional and gene expression analysis resolves heterogeneity within stem cell populations. Cell Stem Cell.

